# Osmotic Pressure and Its Biological Implications

**DOI:** 10.3390/ijms25063310

**Published:** 2024-03-14

**Authors:** Songjie Zheng, Yan Li, Yingfeng Shao, Long Li, Fan Song

**Affiliations:** 1 State Key Laboratory of Nonlinear Mechanics, Beijing Key Laboratory of Engineered Construction and Mechanobiology, Institute of Mechanics, Chinese Academy of Sciences, Beijing 100190, China; 2 Center of Materials Science and Optoelectronics Engineering, School of Engineering Science, University of Chinese Academy of Sciences, Beijing 100049, China

**Keywords:** osmotic pressure, cell division, cell differentiation, lipid phase separation

## Abstract

Gaining insight into osmotic pressure and its biological implications is pivotal for revealing mechanisms underlying numerous fundamental biological processes across scales and will contribute to the biomedical and pharmaceutical fields. This review aims to provide an overview of the current understanding, focusing on two central issues: (i) how to determine theoretically osmotic pressure and (ii) how osmotic pressure affects important biological activities. More specifically, we discuss the representative theoretical equations and models for different solutions, emphasizing their applicability and limitations, and summarize the effect of osmotic pressure on lipid phase separation, cell division, and differentiation, focusing on the mechanisms underlying the osmotic pressure dependence of these biological processes. We highlight that new theory of osmotic pressure applicable for all experimentally feasible temperatures and solute concentrations needs to be developed, and further studies regarding the role of osmotic pressure in other biological processes should also be carried out to improve our comprehensive and in-depth understanding. Moreover, we point out the importance and challenges of developing techniques for the in vivo measurement of osmotic pressure.

## 1. Introduction

Osmosis is a natural process through which solvent molecules move via a semi-permeable membrane to a high solute concentration region from the portion with a low solute concentration, until an equal solute concentration on both sides is achieved [1,2,3,4]. Osmotic pressure is the corresponding pressure required to prevent the net movement of solvent molecules across the semi-permeable membrane, i.e., to nullify the process of osmosis [5,6,7,8,9]. The solutions can be classified into three types: hypertonic, isotonic, and hypotonic, depending on the relative concentration of the dissolved solutes on either side of the semi-permeable membrane [10,11,12,13,14,15]. Specifically, an isotonic solution has the same solute concentration and osmotic pressure as the solution it is being compared to, while a hypotonic solution has a lower solute concentration and osmotic pressure than the solution it is being compared to. On the contrary, a hypertonic solution has a higher solute concentration and osmotic pressure than the solution it is being compared to. It has been well recognized that osmotic pressure is involved in various pathological and physiological processes [16,17,18,19,20,21,22,23,24]. For example, the isotonic solution is a necessary condition for the appropriate exchange of material between cells and their environment and is required for red blood cells to perform their function of delivering nutrients and oxygen. A cell will undergo shrinking and swelling when cultured in the hypertonic and hypotonic solution, respectively [25,26,27,28,29]. Especially, red blood cells rupture and release hemoglobin in response to a hypotonic solution with sufficient low osmotic pressure, which in turn leads to hemolysis and multiple diseases [30,31,32]. Quantitatively, the red blood cells hemolyze when the extracellular osmolarity decreases by about 50% [33]. Actually, the osmolarity of human blood under physiological conditions varies within ±10% [34], thus providing a stable osmotic environment. In addition, animal cells become round from an essentially flat state, a critical process for ensuring a successful cell division, as they enter mitosis [35,36,37,38]. This drastic shape change depends strongly on the osmotic pressure difference between the inside and outside of cells, and its failure will perturb tissue homeostasis and development and contribute to cancer progression [16,39,40,41,42,43,44]. The disturbances of the osmotic pressure of the human body are intimately bound up with the progression of many diseases, such as hyponatremia, hypernatremia, hyperglycemia, osteoarthritis, nephropathy, and muco-obstructive lung diseases [45,46,47]. Meanwhile, therapeutic methods established based on the osmotic effect have made a great contribution to the disease treatment. For example, dialysis, a type of therapy that removes metabolic waste products and excess fluid from the blood, making use of osmotic pressure, has become a standard treatment for patients suffering from kidney failure [45,48]. In addition, the acute osmotic effect of urea has also been utilized in treating the cerebral edema [45,49,50]. Therefore, gaining insight into the osmotic pressure is of great importance for understanding cellular activities and for developing novel avenues against diseases.

At present, two important concerns in the field of osmotic pressure, including its quantitative description [2,51,52,53,54,55] and its effect on cellular activities [21,56,57,58,59,60,61], are being intensively investigated. In this review, we summarize the progress of theoretical research on how to quantitatively describe the osmotic pressure. We present a summary of representative models and equations including the van ‘t Hoff equation and Morse equation for ideal dilute solutions, the virial expansion model for non-ideal dilute solutions, as well as the van Laar equation and free solvent model for non-ideal concentrated solutions. The application and limitation of these models and equations are discussed. Subsequently, we present the advancement on the significant role of osmotic pressure in various biological processes, focusing on the cell division, cell differentiation, as well as lipid phase separation. The relevant mechanisms underlying the impact of osmotic pressure on these biological processes are provided. Meanwhile, we point out some important unsolved issues in these studies. Further, we discuss possible future research directions, focusing on theoretical description and biological implications.

## 2. Theories of Osmotic Pressure

Providing a quantitative description of osmotic pressure is one of the most important goals within this research field. Over the past century, tremendous efforts have been devoted to establishing quantitative relationships between osmotic pressure and various properties of solutions. Several representative theoretical advancements are summarized as follows.

### 2.1. Ideal Dilute Mixtures

#### 2.1.1. van ‘t Hoff Equation

The van ‘t Hoff equation, originally developed by Jacobus Henricus van ‘t Hoff, is a pioneering formula [2,62,63] for quantitatively determining the relationship between osmotic pressure and solute concentrations in diverse solution systems:

(1) ∏=RTc

Here, ∏ represents the osmotic pressure of the solution, *R* the gas constant, *T* the absolute temperature, and *c* the molar concentration of the solute. This equation indicates that the osmotic pressure of a solution is directly proportional to the solute concentration and absolute temperature. Moreover, it exhibits the colligative property that the osmotic pressure solely depends on the number of solute particles and is independent of their chemical identity [64,65,66]. The establishment of the van ‘t Hoff equation has significant importance in, on the one hand, offering a theoretical foundation for the quantitative description of osmotic pressure and, on the other hand, providing insights into the impact of solute on the colligative properties of solutions. 

#### 2.1.2. Morse Equation

Based on the van ‘t Hoff equation, Robert S. H. Morse proposed an equation that relates the ideal osmotic pressure to the molality of the solute *m* [52]

(2) ∏=RTm

taking into account the fact that the solute molecules have a certain size. In comparison with the van ‘t Hoff equation, which is valid for only very dilute solutions, the Morse equation can be applied for dilute solutions with slightly higher concentrations. More specifically, it has been estimated that the van ‘t Hoff equation is not applicable for solutions with a concentration over 0.2 mol/m^3^ due to the significant deviation from experimental data. In contrast, the Morse equation can be still reasonably accurate to at least 0.5 mol/m^3^ [67].

Both the van ‘t Hoff equation and Morse equation have been frequently employed to measure the osmotic pressure of different types of solutions [68,69,70]. However, there is an assumption that there is no interaction between the solute and solvent (ideal solution) underlying the van ‘t Hoff equation and Morse equation. This leads to some inevitable errors when calculating the osmotic pressure of real solutions using the van ‘t Hoff equation and Morse equation [71,72]. For example, Salmani [68] utilized the van ‘t Hoff equation to determine the osmotic pressure of different binary liquid mixtures at various compositions and temperatures but found a significant deviation of van ‘t Hoff results from experimental data especially at higher solute concentrations. A similar discrepancy is also observed when measuring the semipermeable membrane behavior of engineered clay barriers using the van ‘t Hoff equation [70]. In general, the osmotic coefficient $\phi =\prod /\prod_{\mathrm{ideal}}$ is used to characterize the deviation of the measured osmotic pressure of real solutions from that of ideal solutions [55,73]. The osmotic coefficient can be defined by $\phi =(\mu_{A}^{*}-\mu_{A})/RTM_{A}\sum_{i}m_{i}$ and $\phi =-(\mu_{A}^{*}-\mu_{A})/RT\ln(x_{A})$ in terms of molality *m* and mole fraction *x*, respectively. Here, $\mu_{A}^{*}$ and $\mu_{A}$ denote the chemical potential of the pure solvent *A* and the solvent in a solution, respectively. *M_A_* represents the molar mass of solvent *A*. The osmotic coefficient, usually applied to determine the actual osmotic pressure of a solution, can be calculated using various models. For example, according to the Gibbs–Duhem equation, Moggia et al. [73] employed the pseudo-lattice approach to calculate the osmotic coefficients of both symmetric and asymmetric electrolyte solutions from the mean activity coefficient *γ* via $\phi =1+(1/m)\int_{0}^{m}md(\ln\gamma )$. They found a good agreement between their theoretical prediction and the experimental data for different electrolyte solutions, thus providing an effective means to determine osmotic pressure. Moreover, the relationship $\phi =1+(1/m)\int_{0}^{m}md(\ln\gamma )$ provides a way to calculate the activity coefficient of the solute.

### 2.2. Non-Ideal Dilute Solutions

#### Virial Expansion Model

For non-ideal dilute solutions involving solute–solute interactions, the virial expansion method provides a feasible means to determine the osmotic pressure. In an analogy to the case of real gases, this model expresses the osmotic pressure of a solution as a power series of solute concentration *c* [53]

(3) $$\frac{\prod }{RT}=A_{1}c+A_{2}c^{2}+A_{3}c^{3}+\cdots$$

with *A*_i_ (i = 1, 2, 3…) being the virial coefficients. The first virial coefficient *A*_1_ = 1, which enforces that the equation reduces to the van ‘t Hoff equation for an ideal dilute mixture without solute–solute interactions [74,75]. The higher-order osmotic virial coefficient reflects higher-order solute–solute interactions [76,77]. The second virial coefficient *A*_2_ is a measure of the two-body solute–solute interaction, the third virial coefficient *A*_3_ accounts for the ternary solute interaction, etc. [75,76]. In general, positive values of *A*_2_ indicate repulsive solute–solute interactions in a solution, and negative values reflect attractive interactions between solutes [78]. Determining the virial coefficients is the key to allow one to calculate osmotic pressure using the virial coefficient method. Extensive studies have been devoted to establishing the virial coefficients, and the knowledge of virial coefficients has also been tabulated for different solutions. Peyrovedin et al. [79] utilized the Exponential-6, Kihara, as well as hard-core Exponential-6 intermolecular potential functions to compute the second osmotic coefficients and other properties (e.g., theta temperature and average molecular weight) of numerous polymer solutions and polymers. Their results showed that, compared to the other two models, the hard-core Exp-6 model with the lowest average errors has a greater potential to be applied for determining the thermodynamic parameters of polymer solutions [79]. To more accurately describe the osmotic behavior of solutions with many-body interactions, a higher-order virial coefficient should be considered. Caracciolo et al. [80,81] determined third and fourth virial coefficients of polymer solutions using theoretical analysis coupled with Monte Carlo simulations. The accuracy of their results is validated by a comparison with experimental data and field-theoretical predictions, thus providing an alternative method for calculating the higher-order virial coefficient and osmotic pressure of non-ideal dilute solutions. Recently, Mussel et al. [82] estimated the second and third virial coefficients of different monovalent salts solutions. They found that both second and third virial coefficients decrease with increasing calcium concentrations, indicating that the volume transition results from the decreased virial coefficient in response to the addition of Ca^2+^ ions into NaCl solutions [82]. The virial expansion model is an important improvement in respect to the osmotic pressure measurement since the virial coefficients are directly related to the interaction potentials between solutes in solutions. However, if the virial expansions are truncated to a few terms, this model often fails to calculate the osmotic pressure for the full range of concentrations, even for dilute solutions. In addition, the solute–solvent interaction, which may be a dominant factor in many cases, is not considered in the virial expansion model. This also limits the application of the virial expansion model.

### 2.3. Non-Ideal Concentrated Solutions

#### 2.3.1. van Laar Equation

Based on the chemical potential of species in solutions, van Laar proposed a logarithmic equation [51,71]:

(4) $$\prod =-\frac{RT}{V_{0}}\ln a_{1}$$

Here, the activity of the solvent in solution $a_{1}=\gamma_{1}x_{1}$ is a function of activity coefficient $\gamma_{1}$ and mole fraction *x*_1_ of this solvent. *V*_0_ represents the molar volume of the solvent. 

The van Laar equation provides an alternative method for measuring osmotic pressure of the non-ideal concentrated solutions. Hamdan et al. [83] adopted the van Laar equation to study the osmotic behaviors of binary and ternary aqueous solutions and calculated their osmotic pressure according to the water activity obtained from experimentally measured relative humidity. Comparisons of the osmotic behavior between ternary and binary systems revealed positive or negative osmotic synergy effects. Their findings suggest that the ternary solutions composed of MgCl_2_ and NaCl can be chosen as suitable candidates as draw solutions in view of their significant positive synergy and sustainable operational osmotic pressure performance. Smith et al. [84,85] investigated the osmotic pressure of aqueous electrolyte solutions at ambient conditions using the van Laar equation together with the molecular simulation method and derived an optimal force field to model the molecular interactions for the simulation algorithm. In addition, the van Laar equation can also be used to calculate the activity of the solvent in solutions as well. More recently, Hosseni and Ashbaugh established a framework for mapping the simulation results of osmotic force balance to the determinations of water activity [68].

#### 2.3.2. Free Solvent Model

As mentioned above, the solute–solvent interactions should be carefully considered when analyzing the osmotic pressure for the non-ideal solutions. For example, water and many kinds of ions can bind with proteins in physiological media, known as protein hydration [86]. Yousef et al. [54] developed a free solvent model with consideration of the solute–solvent interactions:

(5) $$\prod =-\frac{RT}{V_{0}}\ln a_{1}$$

Here, the activity of the solvent in solution $a_{1}=\gamma_{1}x_{1}$ is a function of activity coefficient $\gamma_{1}$ and mole fraction *x*_1_ of this solvent. *V*_0_ represents the molar volume of the solvent. For an ideal solution with activity coefficient $\gamma_{1}=1$, the free solvent model becomes the van Laar equation [71]. In the free solvent model, the protein with all associated ions and water is treated as a single hydrated macromolecule. The mole fraction of solvent *x*_1_ can be expressed as

(6) $$x_{1}=\frac{N_{1}-\sum_{j=2}^{p+1}v_{1j}N_{j}}{N_{*}+\sum_{j=2}^{p+1}N_{j}}$$

assuming the solutions are composed of *p* proteins and *n* distinct species. Here, $N_{1}$ is the mole of the solvent in the solution, and $N_{*}=N-\sum_{i=1,i\neq 2,p}^{n}\sum_{j=2}^{p+1}v_{ij}N_{j}-\sum_{j=2}^{p+1}N_{j}$ is the final total moles of free solvent after protein–solvent interactions, with $N=\sum_{i=1}^{n}N_{i}$ being the initial total moles of the solution and *v_ij_* being the mole number of component *i* that binds with protein *j* to form the hydrated ones. Yousef et al. [54] adopted this free solvent model to determine the osmotic pressure of concentrated IgG solutions under physiological conditions. Their theoretical predictions of osmotic pressure agree well with the experiment data, even at the highest protein concentration, and show better precision than that of the virial expansion model. The free solvent model has been applied successfully to predict the osmotic pressure of solutions with different globular proteins at moderate salt concentrations [54,87,88,89]. In addition, the free solvent model has also been used to determine the other properties of solutions. For example, McBride and Rodgers adopted the free solvent model to estimate the second virial coefficient, which is related to solute–solvent interactions, ion binding, and hydration. Their results provide interpretations regarding the negative second virial coefficients of solutions with non-attractive proteins [90]. Note that despite the progress made in the free solvent model, it is not entirely applicable to all experimentally feasible temperatures and solute concentrations [71].

## 3. Roles of Osmotic Pressure

### 3.1. Impact of Osmotic Pressure on the Symmetry of Cell Division

The cell membrane is composed of various lipids and proteins [91,92,93] and acts as a protective barrier and gatekeeper [94,95]. The cell membrane is a representative semi-permeable membrane that allows certain ions or molecules to pass through it via osmosis but not others [96]. Gases such as oxygen and carbon dioxide [97] and small polar molecules, such as water [98,99] and ethanol [100], can diffuse across the cell membrane. By contrast, highly charged molecules and large molecules, such as amino acids and sugars cannot cross the membrane via osmosis, and their passage depends on specific transport proteins in the cell membrane [101,102]. The semi-permeable property of cell membranes can lead to the osmotic pressure difference between the interior and exterior of the cell, which is important for various cellular processes. Specifically, during mitosis, there is more than a 10-fold change in the osmotic pressure difference, accompanied with drastic variations in the shape of mitotic cells [103]. It has been recognized that the osmotic pressure is involved in regulating many aspects of mitosis. Here, we focus on the significant role of osmotic pressure in the symmetry of cell division.

**Figure 1:**
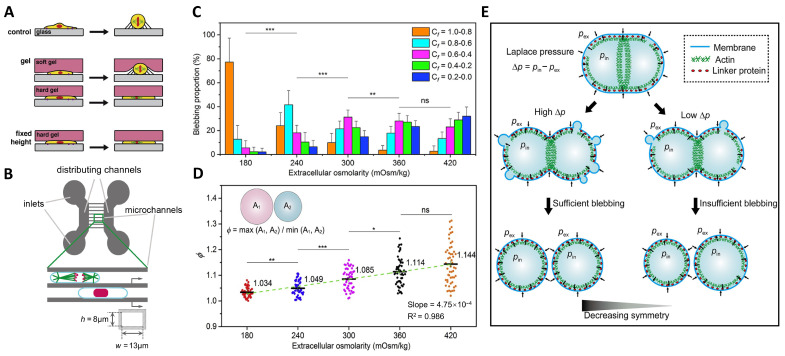
Impact of osmotic pressure on the symmetry of cell division. (**A**) Schematic diagram showing the different physical confinement methods used to perturb mitotic cell rounding (PMDS: polydimethylsiloxane). Reprinted with permission from Ref. [116]. Copyright © 2013 Elsevier Inc. (**B**) Scheme of cells confined in microchannels with height *h* = 8 μm and width *w* = 13 μm. The two large distributing channels are used both to inject the cells and as reservoirs of media. Adapted with permission from Ref. [117]. Copyright © 2018, The Author(s). (**C**) Blebbing proportions and distributions in different stages of the cells during cytokinesis in response to different extracellular osmolarities. The cytokinesis factor *C_f_* = (*r*/2)(1/*R*_1_ + 1/*R*_2_) characterizes the real-time shape of a cell. Here, *r* is the radius of the cleavage furrow, *R*_1_ and *R*_2_ are the effective radii of the two daughter cells. * *p* < 0.05 ** *p* < 0.01; *** *p* < 0.001; ns, not significant. (**D**) Ratio of the projected areas of two daughter cells *ϕ* as a function of extracellular osmolarity. (**E**) Laplace pressure as a function of osmotic pressure difference regulates the symmetry of cell division by way of blebs. Reprinted with permission from Ref. [41]. Copyright © 2021, The Author(s).

During mitosis, a cell can divide symmetrically to produce two daughter cells with an identical size and genetical properties for cell proliferation and self-renewal or asymmetrically to generate two daughter cells with a distinct size and fate for cell diversity and tissue development [104,105,106]. It is generally believed that the mitotic spindle guides the formation, positioning, and orientation of the central furrow to determine the size of two daughter cells and the symmetry of cell division [107,108,109,110,111]. Recent studies revealed that the cellular shape takes part in regulating the symmetry of cell division. It is commonly observed that the adherent cells with spread or elongated morphology in interphases become round and spherical upon entry into mitosis [112,113,114,115]. In the case of cell adhesion to the artificial substrate, the classical focal adhesions disassemble at mitotic entry. However, cell rounding is not simply a result of the loss of cell–substrate adhesion but depends on other driving forces. To measure and identify the forces required for mitotic cell rounding, various technologies and methods, such as micropipettes, atomic force microscopy (AFM), optical stretcher, and acoustic microscopy, have been applied. For example, Stewart et al. [35] used AFM technology to track the cell rounding force in mitosis and found a significant increase in rounding pressure up to 0.14 nN μm^−2^. Their results indicate that the rounding force is generated by an osmotic pressure and thus depends strongly on the cell’s ability to regulate its own osmolarity through controlling ion transporters. As a result of the osmolarity being higher inside the cell than outside, the water will flow into the cell to generate hydrostatic pressure and leads to the cell swelling and rounding. Furthermore, their findings show that the osmotic pressure in cells is balanced by the contractile tension of the actomyosin cortex [35]. Accumulating evidence indicates that the osmotic pressure-regulated mitotic cell rounding plays an important role in controlling the symmetry of cell division. On the one hand, the mitotic rounding of a cell within a crowded environment can generate space to favor the formation of a bipolar spindle. Lancaster et al. [116] performed a series of experiments to regulate mitotic rounding by applying external physical confinement (Figure 1A). Their findings show that limiting mitotic cell height to perturb or even prevent cell rounding leads to defects in spindle assembly and spindle pole splitting, which can cause the formation of multipolar spindles and contribute to an asymmetric cell division. Especially, pole splitting is frequently observed, creating ectopic poles without centrioles, for mitotic cells confined to 8 μm or less in height. This suggests that minimal space is required for a spindle to maintain its bipolarity. They attributed the spindle pole splitting under conditions of physical confinements to the limited reach of microtubules [116]. On the other hand, mitotic cell rounding has been shown to be essential for accurately positioning the spindle, which in turn determines the symmetry of cell division by positioning the cleavage furrow at cytokinesis. The mitotic spindle in a rounded cell is positioned in the cell center achieved via the balance of pulling forces by astral microtubules extending outward from the opposite spindle poles. By contrast, the mitotic spindle in a confined cell mispositions in the cell, giving rise to uneven cell divisions. Cadart et al. [117] confined the HeLa and aneuploid epithelial cells in a narrow microchannel to prevent mitotic cell rounding (Figure 1B). They found that such physical confinements can lead to errors in the positioning of mitotic spindle and ultimately result in an asymmetrical division of the mother cell. Mitotic cell rounding is believed to favor the symmetric position of the spindle and cleavage furrow through facilitating the interactions between the spindle and cortex in all directions, while the external physical confinement elongates the cellular axis and makes the cortex go beyond the reach of astral microtubules in every direction, leading to spindle misplacement and an asymmetrical cell division [118].

Recently, we found another way by which osmotic pressure regulates the symmetry of cell division during cytokinesis [41,119]. We adjusted the extracellular osmolarity to study the effect of the osmotic pressure difference across the cell membrane on division symmetry during the final step in cell division. We found that the osmotic pressure difference controls cell blebbing to regulate the symmetry of cell division. More specifically, making a change to the osmotic pressure difference varies the distribution and size of cell blebbing: the lower the extracellular osmolarity, the greater the proportion of the blebbing in the earlier stage and the sooner the blebbing activity is complete (Figure 1C). Meanwhile, it is found that the lower the extracellular osmolarity, the more symmetric the newborn daughter cells (Figure 1D). We further demonstrated that the symmetry of cell division and cell blebbing, both regulated by the osmotic pressure difference, are intrinsically connected. Our results showed that the cortical structure and the uniformity of the cortex–membrane boundary varied in response to the change in osmotic pressure, which in turn regulates the symmetry of cell division. We proposed a spindle-independent mechanism in which cells rely on osmotic pressure to modify cortex intactness on both sides of the dividing cells during cytokinesis to ensure a symmetric division by way of pressure-driven cell blebbing (Figure 1E). Our findings shed new light on the role of osmotic pressure in ensuring symmetric cell divisions and highlight the significance of pressure-driven blebbing, which has been primarily viewed as a by-product of cytokinesis but now is endowed with new functions.

### 3.2. Impact of Osmotic Pressure on Cell Differentiation

Stem cells are capable of self-renewal and differentiation into varied cell lineages that are fundamental for tissue development, homeostasis, and repair [120,121,122,123,124]. Due to their inherent regenerative capabilities and developmental potency, stem cells have shown great potential in the field of tissue engineering and regenerative medicine and thus have attracted great interest in recent years [125,126,127]. However, an ongoing challenge within the clinical translation of stem cell-based therapeutic paradigms is to control stem cell fate and differentiate them into the desired specific cell types [128,129,130]. It has been well established that stem cells can sense, respond, and adapt to the biological, chemical, and physical stimuli within stem cell niches [123,131] and transduce crucial signals to shape their functions and determine their fate [132]. Tremendous efforts have been dedicated to identifying optimal combinations of cell intrinsic and extrinsic cues to direct stem cell differentiation and function, aiming at developing innovative protocols that can be widely used in basic research and medical applications.

**Figure 2:**
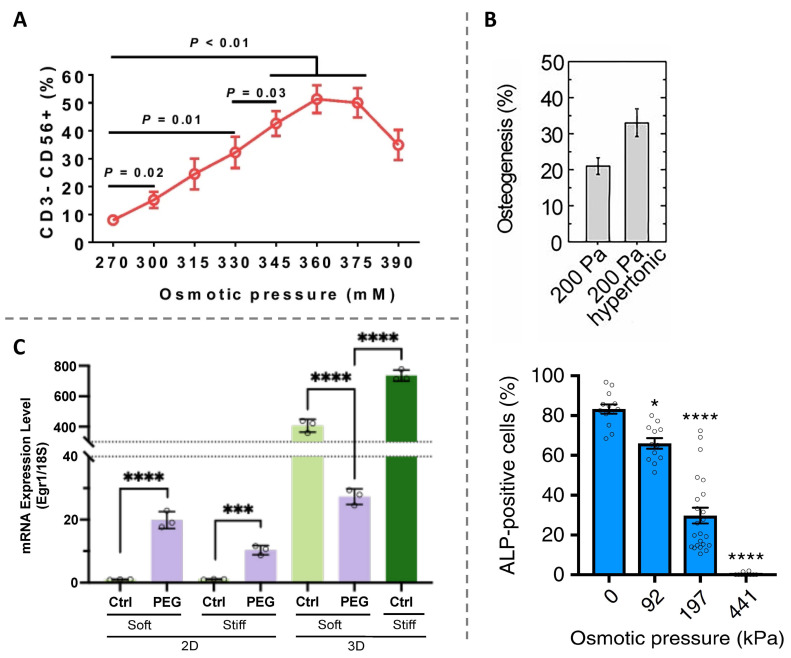
Impact of osmotic pressure on cell differentiation. (**A**) Frequency of CD3^−^CD56^+^ natural killer cell after 14 days of expansion and differentiation of hematopoietic stem and progenitor cells under specific osmotic pressures. Reprinted with permission from Ref. [134]. Copyright © 2023, The Author(s). (**B**) **Top**: percentage of osteogenesis of mesenchymal stem cells grown on 2D substrate (shear modulus of 200 Pa) in response to hyperosmotic pressure. Adapted with permission from Ref. [135]. Copyright © 2017, The Author(s). **Below**: percentage of mesenchymal stem cells stained positive for alkaline phosphatase (ALP, an osteogenic biomarker) encapsulated in 3D microenvironment as a function of osmotic pressure. * *p* < 0.05 and **** *p* < 0.0001. Reprinted with permission from Ref. [136]. Copyright © 2019, The Author(s). (**C**) mRNA expression level of early growth response 1 (Egr1), a mechanosensitive regulator of stem cell lineage commitment, of neural stem cell in response to hyperosmotic pressure by adding 400-Da polyethylene glycol (PEG 400) for the 2D and 3D gels. *** *p* < 0.005 and **** *p* < 0.001. Reprinted with permission from Ref. [138]. Copyright © 2022, The Author(s).

The osmotic pressure has been demonstrated to be associated closely with stem cell differentiation. For example, the differentiation of adipose-derived stem cells (ADSCs) into nucleus pulposus (NP)-like cells is found to be promoted in response to hyperosmolarity below 400 mOsm/kg as quantified via protein and gene analysis. However, further increasing extracellular osmolarity up to 500 mOsm/kg decreases the NP-like gene expression. These findings suggest that there exists a potential optimal osmolarity for successful ADSC differentiation [133]. The histone demethylase KDM4B is shown to respond to the change in extracellular osmolarity and to direct ADSC differentiation into NP-like phenotypes by coordinating with Foxa1/2 [133]. In the case of hematopoietic stem and progenitor cells (HSPCs), increasing the osmotic pressure by adding glucose or sodium chloride into the culture medium can lead to about a ten-fold increase in the efficiency of HSPC differentiation to natural killer (NK) cells that exhibit an enhanced proliferation ability and maintain standard antitumor efficacy (Figure 2A). This finding establishes the extracellular hyper-osmotic pressure as a simple yet powerful protocol to differentiate HSPCs to NK cells [134].

The two-dimensional (2D) cell culture system is the most commonly adopted protocol to study cell biology in vitro in earlier researches and has provided us with enlightening information regarding how the cells sense, respond, and adapt to their surrounding microenvironments. By contrast, the three-dimensional (3D) cell culture system offers a more actual physiological microenvironment. It has been well established that cells cultured in 3D environments differ physiologically and morphologically from those cultured in 2D environments. The additional dimensionality of the 3D culture system not only leads to extra physical constraints placed on cells but also affect the organization and distribution of cell membrane proteins engaged in interactions with surrounding matrices and cells. Growing evidence suggests that osmotic pressure-regulated cell differentiation also shows a confinement dimensionality-dependent manner. Guo et al. [135] reported that osmotic pressure can control the stem cell differentiation pathway (Figure 2B, Top). More specifically, preferential adipogenic and osteogenic differentiations are observed for mesenchymal stem cells (MSCs) under hypotonic and hypertonic conditions, respectively. Their results suggest that intranuclear and intracellular molecular crowding regulated by osmotic pressure has a significant impact on such aspects as protein folding, chromatin structure, and transcription and hence alter stem cell differentiation [135]. However, different response of MSCs to osmotic pressure is found when they are cultured in 3D matrices [136,137]. For example, Lee et al. [136] revealed that hypo-osmotic pressure accelerates the osteogenesis of MSCs restricted in a 3D environment. Instead, the osteogenic commitment significantly diminishes by applying hyper-osmotic pressure (Figure 2B, below). These results are somewhat contrary to the observations for MSCs cultured on 2D substrates. The contrasting results highlight the importance of confining microenvironments in guiding stem cell differentiation and might be partially attributed to the cell–hydrogen interaction. This osmotic pressure-mediated osteogenesis is demonstrated to be the result of the reciprocal feedback between volume expansion and transient receptor potential vanilloid-4 activation, which controls the nuclear localization of runt-related transcription factor 2. Recently, Baek et al. [138] took, for example, neural stem cells (NSCs) to further study the cellular response to osmotic pressure in 2D and 3D microenvironments. Their results indicate that applying hyper-osmotic pressure to inhibit cell volumetric growth and induce confining stress results in a substantial drop in the expression level of early growth response 1 (Egr1), a mechanosensitive regulator of stem cell lineage commitment, in 3D hydrogels, while the opposite result was observed in 2D hydrogels (Figure 2C). This finding helps to explain the difference in the lineage commitment of NSCs in 2D and 3D matrices [138]. Despite these advances in studying the different response of stem cell differentiation to osmotic pressure in 2D and 3D microenvironments, it is still an open issue regarding how the difference in lineage commitment is generated.

### 3.3. Impact of Osmotic Pressure on the Lipid Phase Behavior

Biological membranes are often viewed as a fluid bilayer with a homogeneous distribution of both lipids and proteins as proposed by S. J. Singer and G. L. Nicolson (i.e., fluid mosaic model) 50 years ago [139]. In this fluid mosaic model, the proteins are supposed to diffuse freely within the lipid sea, and the mean square displacement (MSD) of proteins <*r*^2^> depends linearly on their diffusion coefficient *D* and time *t* as <*r*^2^> = 2*Dt* [140]. However, accumulating evidence indicates that the biological membranes are structurally heterogeneous and contain dynamic assemblies with lipid and protein compositions differing from their surrounding membrane. These dynamic assemblies, often termed lipid rafts, exist as liquid-ordered phases partially due to the enriched cholesterol and diffuse freely in their surrounding liquid-disordered matrix of biological membranes [141,142]. The diffusion of proteins within raft domains consisting of more viscous saturated lipids is slower than that within non-raft matrix enriched in unsaturated lipids, which leads to the subdiffusion of proteins following the relationship <*r*^2^> = 2*Dt^α^* with the anomalous diffusion parameter *α* < 1 [140]. The size of the raft unit is smaller than 200 nm and thus is below the resolution limit of conventional optical microscopy. By making use of the lipid–lipid and lipid–protein interactions, the small rafts can coalesce into large phase-separated domains to serve as a signaling platform, which facilitates both the *cis* protein–protein interactions in the same membrane due to the spatial proximity and the *trans* receptor–ligand interactions between opposing membranes attributed to the more configurational entropy of the fluctuating membranes as a result of raft-induced protein aggregation [143,144]. Such liquid–liquid phase separations (LLPSs) of lipids implicate the lipid raft in various physiological and pathological processes, such as signaling transduction and cancer metastasis. Therefore, studying in depth the regulatory mechanism underlying the LLPS of lipids and developing a method to control the LLPS of lipids are of great significance.

**Figure 3:**
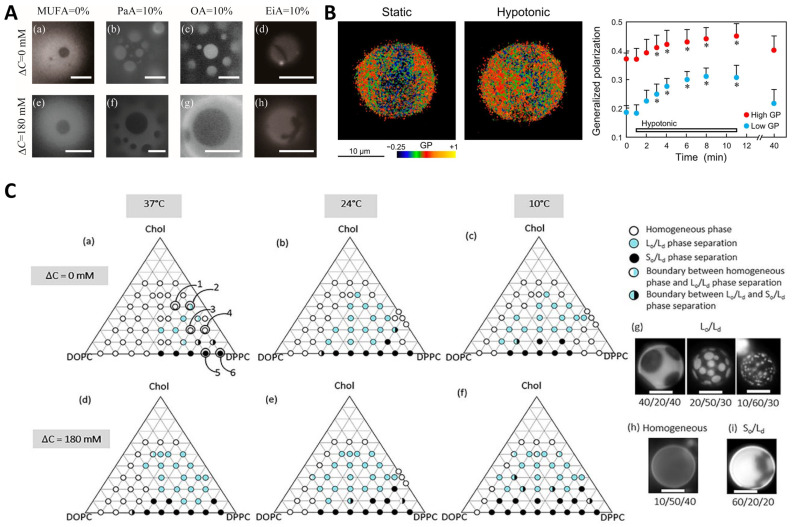
Impact of osmotic pressure on the lipid phase behavior. (**A**) Phase behavior of the lipid membranes containing monounsaturated fatty acids (MUFAs) with different chain lengths: palmitoleic acid (PaA), oleic acid (OA), and eicosenoic acid (EiA) in the absence (**a**–**d**) or presence (**e**–**h**) of hypotonic solution. Scale bars are 5 μm. Reprinted with permission from Ref. [149]. Copyright © 2022, The Author(s). (**B**) Membrane lipid order, as indicated by general polarization (GP) values, in response to hypotonic shock. **Left**: GP images of giant unilamellar vesicle before and 5 min after hypotonic swelling. **Right**: temporal GP changes in both the high- and low-GP regions. * *p* < 0.01. Reprinted with permission from Ref. [147]. Copyright © 2015, The American Physiological Society. (**C**) Schematic phase diagrams (**a**–**f**) and microscopic images of vesicles (**g**–**i**) consisting of dioleoylphosphocholine (DOPC)/dipalmitoylphosphocholine (DPPC)/cholesterol (Chol) with and without hypotonic shock at three temperature levels. DOPC/DPPC/Chol at ratios of 20/40/40, 10/50/40, 20/60/20, 10/70/20, 20/80/0, and 10/90/0 correspond to 1–6 in (**a**), respectively. Scale bars are 10 μm. Reprinted with permission from Ref. [148]. Copyright © 2020, The American Chemical Society.

Osmotic pressure has been shown to be involved in regulating the LLPS of lipids. Giant unilamellar vesicles (GUVs) are frequently used to study the response of phase behavior to osmotic pressure. GUVs swell or shrink when exposed to the hypotonic or hypertonic medium, accompanied with the change in membrane tension (Figure 3A). The osmotic tension has been demonstrated to facilitate raft domain formation and induce phase separation (Figure 3B) [145,146,147,148,149,150,151,152,153]. Wongsirojkul et al. [148] studied the osmotic-induced phase separation of vesicles composed of dipalmitoylphosphocholine (DPPC), dioleoylphosphocholine (DOPC), as well as cholesterol (Chol) and constructed the ternary phase diagram at different temperature levels (Figure 3C). They found that the osmotic tension enhances the line tension by evaluating the domain boundary fluctuation and contributes to the phase separation, depending strongly on the cholesterol content in membranes. Further, they showed that osmotic tension can greatly shift the miscibility temperature via fluorescence microscopy. Similarly, Emami et al. [150] found that tense membranes of GUVs suffering a proper hypo-osmotic shock promote the lipid phase separation into coexisting sterol-enriched liquid-ordered and sterol-depleted liquid-disordered domains. By contrast, the situation may be different for real cells. Colom et al. [151] reported that after a hyper-osmotic shock, the membrane composition in Hela cells becomes more homogeneous as a result of the decreased membrane tension, which is consistent with the observation for the GUV with a lipid mixture DOPC:sphingomyelin:Chol in a molar ratio of 1:1:1. However, Riggi et al. [153] investigated the activation of the target of rapamycin complex 2 (TORC2) in response to the hypo- and hyper-osmotic shocks and found that TORC2 senses the increased and decreased tension of cell membranes through different mechanisms. Particularly, following hyper-osmotic shock, the decreased membrane tension triggers the lipid phase separation within the yeast cell membrane and the redistribution of phosphatidylinositol-4,5-bisphosphate (PtdIns(4,5)P_2_) into invaginated membrane domains, which in turn inactivate TORC2. The difference regarding the effect of osmotic pressure on the lipid phase separation as obtained by Riggi et al. [153] and Colom et al. [151] may attribute to the different cell types, e.g., there is a cell wall outside the yeast cell membrane, while there is not for Hela cells. These findings by Riggi et al. suggest that protein sorting via lipid phase separation provides an additional regulatory mechanism for signal transduction [153].

Recently, our results indicated that membrane adhesion via the *trans* interactions between receptors and ligands that are associated with lipid rafts can reduce the threshold value of raft–raft contact energy required for phase separation, i.e., facilitate the LLPS [154,155,156,157,158,159,160]. This promotion results from the thermal fluctuations of the opposing membranes. The regulatory mechanism is as follows: The thermal membrane fluctuations lead to an effective attraction between receptor–ligand complexes so that the flexible membranes can access more configurations. By virtue of the association of lipid rafts with these adhesion proteins, these rafts tend to aggregate to form large domains. A change in the osmotic pressure difference across the membrane can tense or relax this membrane [161,162,163,164], subsequently suppressing or enhancing thermal membrane fluctuation, which in turn affects the lipid phase behavior. Further, we found that the LLPS enhances the binding constant and binding cooperativity of membrane-anchored receptors and ligands due to the induced aggregation of these adhesion proteins [144,155,162,165]. The thermal membrane fluctuation makes a positive contribution to the receptor–ligand interactions in the adhesion system with raft-containing membranes, in sharp contrast to that with homogeneous membranes. Our findings further highlight the important role of lipid phase separation in signal transduction and expand the potential function of osmotic pressure.

## 4. Conclusions and Future Prospectives

Osmotic pressure depends strongly on the solution properties and has been involved in numerous fundamental biological activities across scales, from molecular interactions to cellular processes, to organ development and tissue homeostasis. Gaining insight into osmotic pressure and its effect on biological activities will contribute to both biomedicine and pharmaceutical fields. In this review, we summarized the advancements regarding the theoretical description of osmotic pressure and the important roles of osmotic pressure in cell division, cell differentiation, as well as lipid phase separation. More specifically, we discussed different theoretical equations and models including the van ‘t Hoff equation and Morse equation for ideal dilute mixtures and the virial expansion model, the van Laar equation and free solvent model for non-ideal solutions, with particular emphasis on their applicability and limitations. Further, we discussed the osmotic pressure dependence of LLPS, cell division and differentiation, focusing on the mechanisms underlying the effect of osmotic pressure on these biological processes. These studies not only enrich our understanding of osmotic pressure and its significant roles but also provide potential protocols and interventions for therapeutic development and preventative treatments.

Note that despite the substantial progress made to data, on the one hand, theoretical models to quantitatively describe the osmotic pressure for all experimentally feasible temperatures and solute concentrations are still lacking, and on the other hand, it is still an open issue regarding how osmotic pressure affects biological activities across scales. In practice, we usually regulate extracellular osmolarity to study the cellular response and cannot actually establish the direct and quantitative relationship between osmotic pressure and its biological implications. The theoretical advancement in the studies of osmotic pressure will contribute to solving this issue. In addition, measuring osmotic pressure in vivo remains very challenging despite the fact that much effort has been made [166]. Developing techniques for the measurement of osmotic pressure inside organelles, cells, or tissues will no doubt facilitate our understanding of the roles that osmotic pressure plays in living organisms and contribute to opening new avenues for therapeutic intervention.
